# C-myc/miR-150/EPG5 axis mediated dysfunction of autophagy promotes development of non-small cell lung cancer

**DOI:** 10.7150/thno.34887

**Published:** 2019-07-09

**Authors:** Hui Li, Juan Liu, Wenjie Cao, Xiaojuan Xiao, Long Liang, Feng Liu-Smith, Weiwei Wang, Hong Liu, Peng Zhou, Ruoyun Ouyang, Zhijun Yuan, Jing Liu, Mao Ye, Bin Zhang

**Affiliations:** 1 Molecular Biology Research Center & Center for Medical Genetics, School of Life Sciences, Central South University, Changsha 410078, China.; 2 Department of Histology and Embryology, Xiangya School of Medicine, Central South University, Changsha 410013, China.; 3 The First Xiangya Hospital, Central South University, Changsha 410078, China.; 4 Department of pathology, The Second Xiangya Hospital, Central south university, Changsha, 410011, China.; 5 Department of Respiratory Medicine, Respiratory Disease Research Institute, The Second XiangYa Hospital, Central South University, Changsha, 410011, China.; 6 Head & Neck Internal Medicine Area, Hunan Provincial Tumor Hospital, Changsha, 410013, China.; 7 Molecular Science and Biomedicine Laboratory, State Key Laboratory for Chemo/Biosensing and Chemometrics, College of Biology, College of Chemistry and Chemical Engineering, Collaborative Innovation Center for Chemistry and Molecular Medicine, Hunan University, Changsha 410082, China.

**Keywords:** NSCLC, miR-150, EPG5, autophagy, tumorigenesis

## Abstract

**Rationale**: Lung cancer is the leading cause of cancer death worldwide, and treatment options are limited to mainly cytotoxic agents. Here we reveal a novel role of miR-150 in non-small cell lung cancer (NSCLC) development and seek potential new therapeutic targets.

**Methods**: The miR-150-mediated autophagy dysfunction in NSCLC cells were examined using molecular methods *in vitro* and *in vivo*. The upstream regulatory element and downstream target of miR-150 were identified *in vitro* and validated *in vivo*. Potential therapeutic methods (anti-c-myc or anti-miR-150) were tested *in vitro* and *in vivo*. Clinical relevance of the c-myc/miR-150/EPG5 axis in NSCLC was validated in human clinical samples and large genomics database.

**Results**: miR-150 blocked the fusion of autophagosomes and lysosomes through directly repressing EPG5. The miR-150-mediated autophagy defect induced ER stress and increased cellular ROS levels and DNA damage response, and promoted NSCLC cell proliferation and tumor growth. Knockdown of EPG5 promoted NSCLC cell proliferation, and attenuated the effects of miR-150. c-myc gene was identified as a miR-150 transcriptional factor which increased miR-150 accumulation, therefore pharmacologically or genetically inhibiting c-myc/miR-150 expression significantly inhibited NSCLC cell growth *in vitro* and *in vivo*. Both c-myc and miR-150 were significantly over-expressed in NSCLC, while EPG5 was down-regulated in NSCLC. Expression levels of these molecules were well correlated, and also well correlated with patient survival.

**Conclusions**: Our findings suggest that c-myc/miR-150/EPG5 mediated dysfunction of autophagy contributes to NSCLC development, which may provide a potential new diagnostic and therapeutic target in NSCLC.

## Introduction

Lung cancer is the most commonly diagnosed cancer and the leading cause of cancer mortality worldwide 1. As the most frequently diagnosed pathological subtype, non-small cell lung cancer (NSCLC) counts for nearly 80% of all lung cancer cases 2. Despite continuous development of novel therapeutic methods, the overall 5-year survival rates vary from 4-17% depending on the stage and regional differences 3. Further understanding of molecular mechanisms associated with NSCLC progression is urgently needed in order to develop novel and effective therapeutic strategies.

Macroautophagy (autophagy) has been reported as an important regulatory mechanism for tumorigenesis and progression of a variety of cancers, including NSCLC 4, 5. Autophagy is a process of metabolic degradation at the cellular level, in which protein aggregates and defective organelles of the cytosol are sequestered by double-membrane autophagosomes, and then further fused with lysosomes for degradation 6. This complex process is fine-tuned by several mediators 7, 8. Upon the formation of autophagosome, the p62 protein act as a selective autophagy receptor for degradation of ubiquitinated substrates, which directly interacts with LC3 9. Consequently, the LC3 is cleaved by autophagy-related protein 4 (ATG4) to form cellular LC3I, that further covalently conjugated to phosphatidylethanolamine on the phagophore membrane to form LC3II, and finally both p62 and LC3II degraded by fusion with lysosome 10. Therefore, impaired autophagy is accompanied by accumulation of p62, LC3II and ubiquitinated protein aggregates, and the level of p62 and LC3 combined with tandem fluorescent-tagged LC3 (ptf-LC3) has been used as markers for autophagic flux and the lysosomal fusion process 9, 11, 12. Recently, *EPG5* gene has been reported to be essential for mediating the fusion of autophagosomes with late endosomes/lysosomes, in which EPG5 acts as a tethering factor through interaction with LC3 and Rab7 13. Previous study documented that recessive mutations in *EPG5* gene cause a defective autophagy related human multisystem disorder Vici syndrome 14. However, the role of EPG5 in NSCLC and the up-steam regulatory molecules of EPG5 remains unknown.

Increasing evidences have been demonstrated that miRNAs play crucial roles in the regulation of autophagy process through balancing or modulating the expression of the autophagy-related targeting genes 15. For instance, miR-30a can directly bind the 3'-UTR of beclin1 and inhibits beclin1 expression to decrease autophagic activity in tumor cells 16. miR-34a can impair autophagosome-lysosome fusion through repressing autophagy-related protein 9A (ATG9A) expression in cochlear cells 17. In recent years, studies from us and others showed that miR-150 is over-expressed in NSCLC which significantly correlates the clinical TNM stages and poor prognosis 18, 19. Interestingly, similar with miR-150, up-regulation of p62 and LC3 was detected in NSCLC tumor tissues from patients, and a combined high level of LC3 and p62 was significantly associated with aggressive tumor behavior and shorter survival of NSCLC 20, 21. However, whether miR-150 regulates autophagy further contributes to NSCLC development is unknown.

In this study, we demonstrate that miR-150 inhibits autophagic flux, increases cellular ROS level and DNA damage, promotes NSCLC progression through repressing EPG5, which is shown to be a novel tumor suppressor in NSCLC by our evidence. Meanwhile, we found that c-myc increased the transcription and maturation of miR-150, and pharmacologically inhibition of c-myc inhibited NSCLC both *in vitro* and *in vivo*. Furthermore, the high expression of c-myc and miR-150 exhibited significant negative correlation with EPG5 in NSCLC patients. We concluded that c-myc/miR-150/EPG5 axis-mediated dysfunction of autophagy contributed to NSCLC development, suggesting a potentially novel diagnostic and therapeutic target for NSCLC.

## Materials and Methods

### Human lung tissues and cell lines

The human NSCLC tissues and non-neoplastic lung tissues were collected from the Xiangya Hospital of Central South University (ChangSha, China). This study was approved by the Ethics Committee of Xiangya Hospital. The human NSCLC cell lines (A549, H1299, H460, 95C, 95D) and human normal lung cell line (MRC-5) were purchased from the Cell Bank of Type Culture Collection of the Chinese Academy of Sciences, Shanghai Institute of Cell Biology.

### Plasmids and transfection

pSUPER-miR-150 plasmid was constructed by our lab and the transfection experimental protocols were performed according to our previously published protocols 18. The miR-150 inhibitor and the small interfering RNAs (siRNAs) targeting EPG5 and negative control (siNC) were synthesized by GenePharma Co., Ltd (Shanghai, China). The chemically stabilized cholesterol-conjugated miR-150 mimics (named as agomir-150) and miR-150 inhibitor (named as antagomir-150) and each control were obtained from RiboBio Co., Ltd. (Guangzhou, China). The above-mentioned detailed sequences are shown in Table S1. For generation of stable cell lines, pSUPER-miR-150 and EPG5 shRNA-expressing plasmids or control vectors were transfected into NSCLC cells and screened for 3-4 weeks with 1 μg/ml puromycin after a 48 h transfection.

### RNA extraction and quantitative reverse transcription-PCR (qRT-PCR)

mRNA or miRNA expression was measured by qRT-PCR analysis as previously described 18. mRNA expression was normalized to GAPDH and miRNA was normalized to U6 snoRNA. The primers used for mRNAs and miRNA are listed in Table S1**.**

### Western blotting analysis

Western blotting analysis was performed as we previously described 22. Specific antibodies used were summarized in the Table S2.

### Transmission electron microscopy (TEM)

Cells were fixed with 2.5% glutaraldehyde for 24 h, post-fixed with 2% OsO4 for 2 h, followed by dehydration. Thin sections (50 nm) were cut on an Ultramicrotome (LKB-3 microtome, Sweden) and stained with uranyl acetate and lead citrate. Images were visualized by transmission electron microscope (HT7700, Japan).

### Immunofluorescence

Cells were fixed with 4% paraformaldehyde solution for 15 min, washed three times with phosphate-buffered saline (PBS) and then permeabilized cells with 0.1% Triton X-100/PBS for 5 min. The fixed preparations were blocked in 3% BSA/PBS for 1 h, incubated with primary antibodies for 1 h at room temperature, washed three times with PBS and then stained with fluorescence-conjugated secondary antibody (Invitrogen, Carlsbad, MA, USA). Specific antibodies used were summarized in the Table S2. Fluorescent images were acquired by the Leica SP5 II scanning confocal microscope (Leica, Bannockburn, USA).

### Quantitative LC3 puncta analyses

Pri-miR-150 and mCherry-GFP-LC3 were co-transfected into cells for 48 h. Nutrient starvation was induced by treating cells with HBSS for 4 h. Fluorescent images of mCherry-GFP-LC3 were acquired by the Leica SP5 II scanning confocal microscope (Leica, Bannockburn, USA).

### Dual luciferase reporter assay

The human EPG5 wild type or mutated 3'-UTR sequence containing the miR-150 binding site was cloned into the psiCHECK-2 vector. The primer sequences are listed in Table S1. Cells were seeded into 24-well plates and co-transfected with pri-miR-150 or control vector and wild-type or mutated *EPG5* 3'-UTR using Lipofectamine 3000. Both firefly and Renilla luciferase activities were measured after the 48 h transfection using the Dual-Luciferase Reporter 1000 Assay System (Promega, WI, USA) and were detected by the GloMax TM 20/20 detection system (E5331, Promega, WI, USA) according to the manufacturer's instructions. Luciferase activities were normalized to Renilla luciferase.

### MTT assay

Transient or stable transfected cells (3,000/well) were seeded in 96-well plates and grown for 1, 2, 3, 4 or 5 days. Subsequently, 20 µl of MTT working solution was added to the medium and incubated for 4 h after which the medium was removed, and 200 µl of dimethyl sulfoxide was added to dissolve the formazan crystals. The absorbance at 570 nm (A570) was measured on a microplate reader (BioRad, Hercules, USA).

### Colony formation assays

Cells (1000/well) were seeded in 6-well culture plates and each group had 3 wells. After incubation for 14 days at 37℃, colonies were washed three times with PBS and stained with crystal violet solution. The colonies composed of more than 50 cells in a well were counted under a microscope.

### Determination of ROS

Transfected cells were disassociated by trypsinization, washed by DPBS and suspended in HBSS with 10 μM of H2DCFDA (Invitrogen, Carlsbad, MA, USA) for incubating 15 minutes at 37°C. Fluorescence was detected by flow cytometry.

### *In vivo* tumorigenesis in nude mice

*In vivo* experiments were approved by the Animal Care and Use Committee of the third Xiangya Hospital of Central South University (Changsha, Hunan, China). All mice (BALB/C, nu/nu) were 4-6 weeks old, female, and purchased from the Shanghai Lab Animal Research Center (Shanghai, China). For tumorigenicity assays, 2×10^6^ stably transfected with miR-150 or shEPG5 cells in 0.2 ml RPMI-1640 medium were injected subcutaneously into the right upper back of the mice. The tumor volume for each mice was measured every 3 days. Three weeks after the injections, tumor-bearing mice were sacrificed, and the size of the tumor was measured by caliper measurement. Each group had 4-6 mice.

### Treatment experiments in nude mice

All mice (BALB/C, nu/nu) were 4-6 weeks old, female, and purchased from the Shanghai Lab Animal Research Center (Shanghai, China). For treatment with miR-150 agomir or antagomir, 2×10^6^ stably transfected with shEPG5 or shNC cells were injected subcutaneously into the right upper back of the mice. Xenograft tumors were allowed to grow for 4 days and then the animals were divided into four groups. Mice were tail intravenous injected with agomir or antagomir and their control reagent every 4 days, respectively. The tumor volume for each mouse was measured every 3 days. 20 days after the injections, tumor-bearing mice were sacrificed, and the size of the tumor was measured by caliper measurement. For treatment with 10058-F4, AZD5153 or DMSO, 2×10^6^ A549 cells were injected subcutaneously into the right upper back of the mice. Tumors were allowed to grow for 3 days and then the animals were divided into three groups for therapy testing. Mice were intraperitoneal injected with 10058-F4 (20mg/kg), AZD5153 (10mg/kg) or DMSO every 3 days, respectively. The tumor volume for each mouse was measured every 3 days. 20 days after the injections, tumor-bearing mice were sacrificed, and the size of the tumor was measured by caliper measurement.

### Chromatin Immunoprecipitation

Chromatin Immunoprecipitation (ChIP) assay was performed with Pierce™ Magnetic ChIP Kit (Thermo Fisher Scientific, Waltham, MA, USA) following the manufacturer's protocol with some modifications. Briefly, cell suspensions were crosslinked with a final concentration of 1% formaldehyde. Chromatin was isolated, digested by mung bean nuclease (MNase), sheared by sonication, and immunoprecipitated with antibodies. Immunoprecipitated DNA was washed and eluted according to the manufacturer's instructions. Eluted DNA and sheared input material were analyzed by qRT-PCR.

### Immunohistochemical staining

Paraffin sections prepared from clinical NSCLC tumor or adjacent tissues were used for immunohistochemistry assays to detect protein expression levels of c-myc, p62, LC3, γ-H2AX or EPG5 proteins. The indirect streptavidin-peroxidase method was used according to the manufacturer's introduction. The antibodies used were summarized in the Table S2.

### Statistical analyses

All statistical analyses were performed using SPSS 16.0 statistical software. Student's t-test was used to determine the significance of the differences between the control and the experimental groups. A two-sided *p*<0.05 was considered statistically significant.

## Results

### miR-150 inhibited the fusion of autophagosomes with lysosomes in NSCLC

**Figure 1:**
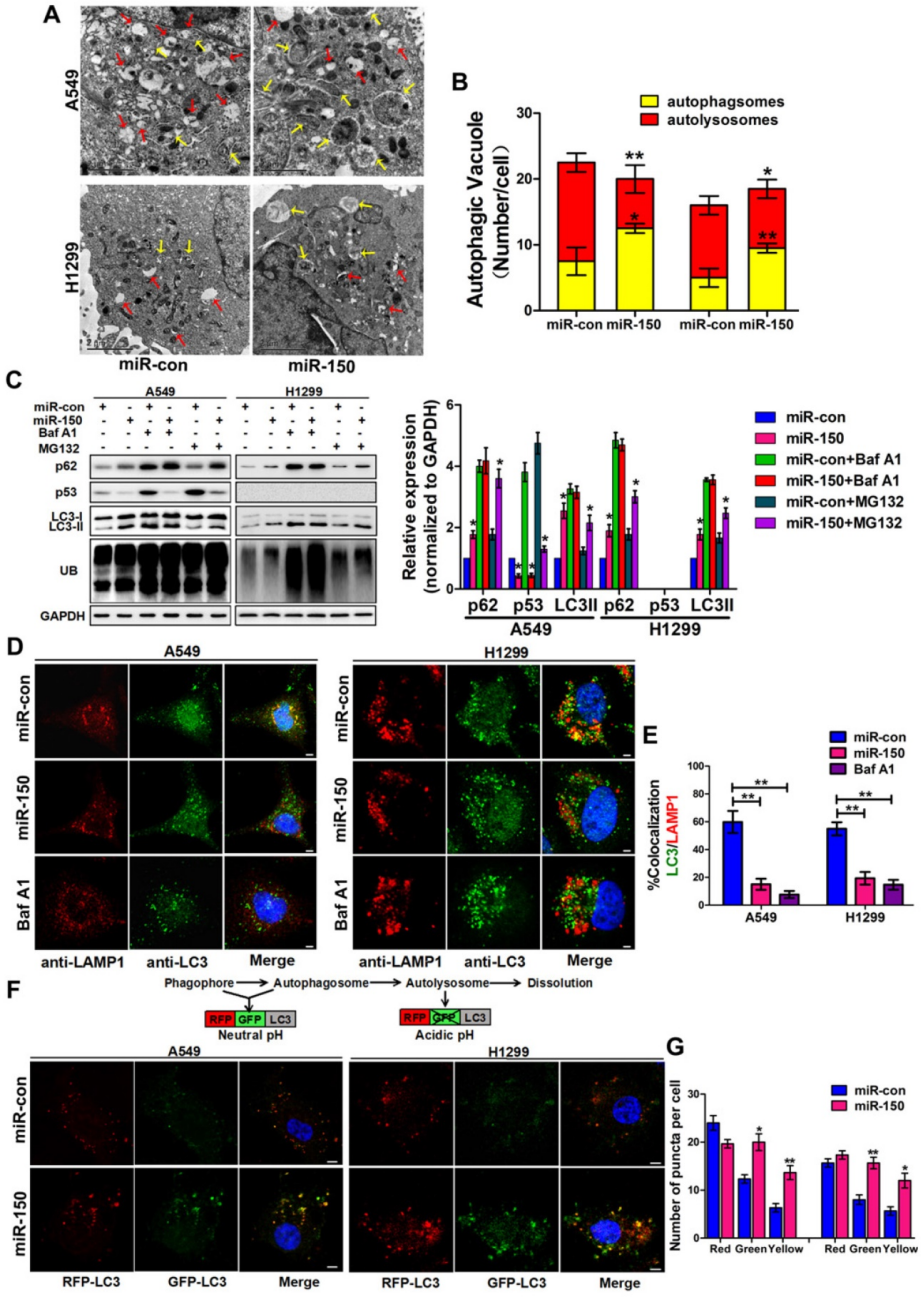
** miR-150 inhibited the fusion of autophagosomes with lysosomes in NSCLC.** (A) Overexpressing miR-150 increased the number of autophagosomes (yellow array) and decreased the number of autolysosomes (red array) in A549 and H1299 cells. Data are representative images of TEMs of three independent assays. Scale bar, 2 μm. (B) Quantification of autophagosomes and autolysosomes per cell are expressed as mean±SEM, n= ~20 to 40 cells per group, *p<0.05, **p<0.01. (C) A549 and H1299 cells transfected with miR-150 or control vector were treated with Baf A1 (100 nM) and MG132 (10 μM) for 4 h and indicated proteins were detected by western blotting. Images are representatives and bar graphs are quantified results of three independent experiments expressed as the mean±SEM, *p<0.05 compared to the control. (D) Colocalization of LC3 and LAMP1 was detected by immunofluorescence. Scale bar, 10 μm. Data are representatives of three independent assays. (E) The LC3 and LAMP1 colocalization coefficiency is expressed as mean±SEM, n= ~30 to 50 cells of three independent experiments. (F) A549 and H1299 cells were cotransfected with miR-150 or control vector and mRFP-GFP-LC3. After 48 h, the cells were cultured with HBSS for 4 h and the autophagy flux was detected with scanning confocal microscope. Data are schematic drawing of the autophagy process (upper) and representative microscopy images of three independent assays showing red-coloured autophagolysosomes or yellow-coloured autophagosomes (lower). Scale bar, 10 mm. (G) Quantification of red, green and yellow puncta per cell are expressed as mean±SEM, n= ~20 to 40 cells. *p<0.05, **p<0.01.

To investigate the role of miR-150 in regulating the autophagy flux, we transfected miR-150 into NSCLC cells (A549, H460 and H1299). Under the electron microscope, large numbers of autophagosomes and less autolysosomes were observed in miR-150 overexpressed cells, which were similar to the H1299 cells treated with bafilomycin A1 (Baf A1) (Figure 1A-B, Figure S1A), a lysosome inhibitor, that is used to evaluate autophagic flux 23. Meanwhile, we examined the expression of p62, LC3 and ubiquitinated protein aggregates (marked as UB), and found that overexpression of miR-150 increased the protein level of p62, LC3II and ubiquitinated protein aggregates. These miR-150 overexpression-enhanced effects were inhibited by Baf A1, but not by proteasome inhibitor MG132 (Figure 1C, Figure S1B), suggesting that miR-150 interfered with the substrate clearance by compromising autophagy/lysosome pathway rather than ubiquitin-proteasome system (UPS). In order to examine whether the increased p62 was due to an indirect transcriptional upregulation, we measured p62 transcript levels and found no significant changes between control and miR-150 overexpressing cells (Figure S1C), suggesting that the p62 accumulation was not due to transcriptional up-regulation, but perhaps a perturbation in autophagic degradation. Furthermore, immunofluorescence staining revealed that miR-150 overexpressed and Baf A1 treated cells were greatly enriched with autophagosomes labeled by LC3, while the co-localization of LC3 and lysosome-associated membrane protein 1 (LAMP1) were obviously decreased (Figure 1D-E, Figure S1D). To further determine autophagic flux, OPTN (Optic neuropathy-inducing protein, Optineurin), a most recently identified selective autophagy substrate 24, was found to accumulate in miR-150-overexpressed cells, which was inhibited by Baf A1 (Figure S1E). Next, we used a mRFP-GFP-LC3 reporter construct to analyze the autophagosomes and autolysosomes fusion, which can distinguish autophagosomes (mCherry positive/GFP positive; yellow dots) from autolysosomes (mCherry positive/GFP negative; red dots) 11. As shown in Figure 1F-G, miR-150-transfected A549 and H1299 cells showed a decrease number of autolysosomes and an increase number of autophgosomes. Taken together, our results suggested that miR-150 suppressed autophagic flux by inhibiting the fusion of autophagosomes with lysosomes.

### miR-150 induced ER stress, ROS and DNA damage, and promoted NSCLC tumorigenesis

**Figure 2:**
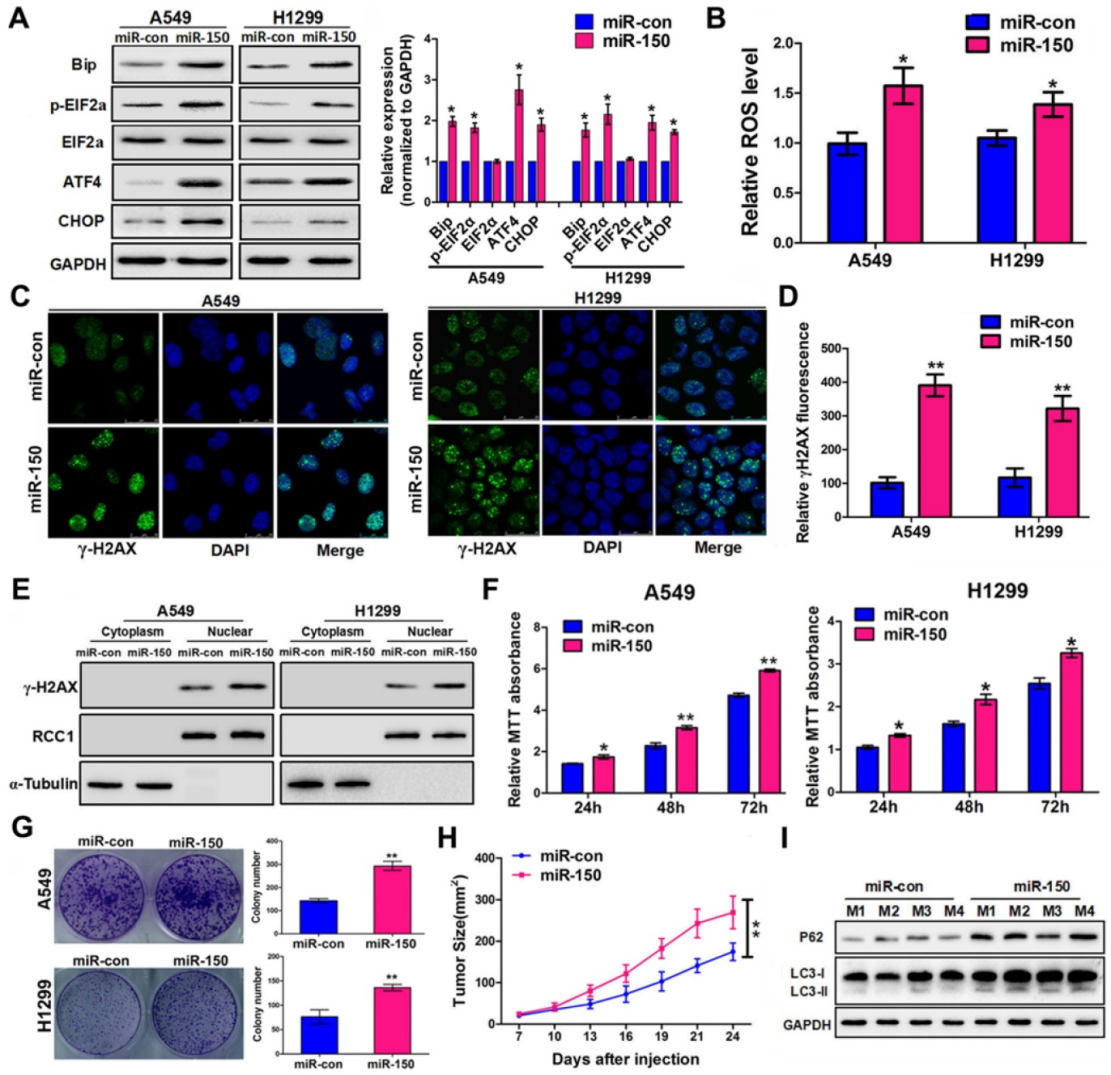
** miR-150 induced ER stress, ROS and DNA damage, and promoted NSCLC tumorigenesis.** (A) A549 and H1299 cells transfected with miR-150 or control vector and ER stress-related proteins were detected by western blotting. Images are representatives and bar graphs are quantified results of three independent experiments expressed as the mean±SEM, *p<0.05 compared to the control. (B) A549 and H1299 cells transfected with miR-150 or control vector for 48 h, and the intracellular ROS levels were detected by flow cytometry. Data are quantified results of three independent experiments expressed as the mean±SEM, *p<0.05. (C, D) The representative images of immunofluorescence of γ-H2AX in A549 and H1299 cells transfected with miR-150 or control vector (C) and the γ-H2AX foci fluorescence intensity was expressed as mean±SEM, n= ~30 to 50 cells of three independent experiments (D). (E) The expression levels of endonuclear γ-H2AX in A549 and H1299 cells transfected with miR-con or miR-150 were determined by western blotting. (F) A549 and H1299 cells proliferation as measured by the MTT assay after transfected with miR-150 or control vector, data are quantified results of three independent experiments expressed as the mean±SEM, *p<0.05, **p<0.01. (G) A549 and H1299 cells clonality as measured by the plate clone formation assay after stably transfected with miR-150 or control vector. Images are representatives and bar graphs are quantified results of three independent experiments expressed as the mean±SEM, **p<0.01 compared to the control. (H) H460 cells stably transfected with miR-150 or control vector were injected subcutaneously into the upper backs of the BALB/c nude mice. Data are mean volumes ±SEM at indicated times (n=4 per group), **p<0.01. (I) The indicated autophagy-related proteins in xenograft tumors were detected by western blotting.

Previous studies have shown that autophagy-defective tumor cells preferentially accumulate ER chaperones, ROS, and damaged DNA lesions 25, 26. We observed an elevated level of ER stress-activating related proteins Bip, p-eIF2α, ATF4 and CHOP in miR-150 overexpressed A549 and H1299 cells (Figure 2A), suggesting that miR-150-mediated autophagy defects induced ER stress. Since autophagy deficiency causes accumulation of damaged mitochondria and induces the oxidative protein folding machinery, ROS levels were examined following miR-150 overexpression. As shown in Figure 2B, A549 and H1299 cells transfected with miR-150 displayed an increase in the ROS level compared to the control cells. Furthermore, immuno- fluorescence revealed that overexpression of miR-150 induced distinct γ-H2AX foci formation in cells (Figure 2C-D). In addition, western blotting showed that overexpression of miR-150 markedly increased the accumulation of endonuclear γ-H2AX (Figure 2E), suggesting that miR-150 increased the DNA damage of NSCLC cells. More importantly, the oncogenic effect of miR-150 was evident by *in vitro* assay and the ability to enhance tumorigenesis was observed* in vivo*. Overexpression of miR-150 enhanced the proliferation of A549, H1299 and H460 cells *in vitro* as evaluated through MTT and plate colony formation assay (Figure 2F-G, Figure S2A-B). On the other hand, subcutaneous tumors from the H460 cells stably overexpressing miR-150 grew faster and had larger tumor volumes than those from the control group (Figure 2H, Figure S2C). In addition, deficient autophagic flux was further confirmed in the miR-150 over-expressed mice tumors (Figure 2I). Collectively, our results suggested that overexpression of miR-150 induced autophagy deficiency and accumulation of ER stress, ROS and DNA damage, and promoted the NSCLC tumorigenesis.

### EPG5 was a novel target for miR-150

**Figure 3:**
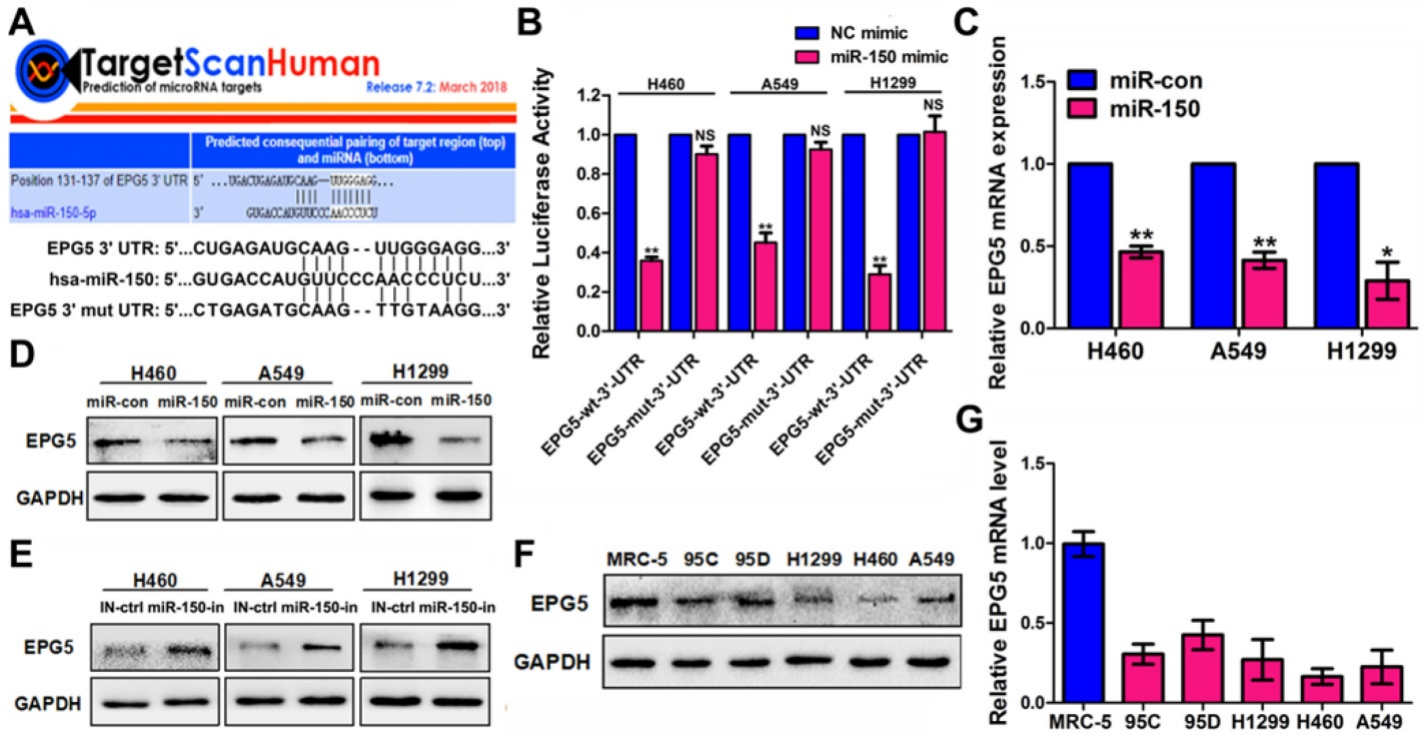
** EPG5 was a novel target for miR-150.** (A) Schematic illustration of the predicted miR-150 conserved binding site in EPG5 3'-UTR of wild-type EPG5 mRNA (EPG5-wt-3'-UTR), and a EPG5 mutant containing two mutated nucleotides in the 3'-UTR of EPG5 (EPG5-mut-3'-UTR). (B) Luciferase activity assays of the EPG5-wt-3'-UTR or EPG5-mut-3'-UTR reporter in H460, A549 and H1299 cells transfected with miR-150 or the control vector. Data are quantified results of three independent experiments expressed as the mean±SEM, **p<0.01, ^NS^p>0.05. (C, D) The mRNA level (C) and protein level (D) of EPG5 in H460, A549 and H1299 cells transfected with the miR-con or miR-150 were measured by qRT-PCR and western blotting, respectively. GAPDH served as control. Images are representatives and bar graphs are quantified results of three independent experiments expressed as the mean±SEM, *p<0.05, **p<0.01. (E) The protein level of EPG5 in H460, A549 and H1299 cells transfected with the miR-150 inhibitor (miR-150-in) or inhibitor control (IN-ctrl) were measured by western blotting. GAPDH served as control. Data are representatives of three independent assays. (F, G) The protein level (F) and mRNA level (G) of EPG5 were measured in a human normal lung cell line MRC-5 and five different NSCLC cell lines (95C, 95D, H1299, H460 and A549) using qRT-PCR and western blotting, respectively. GAPDH served as control. All experiments were performed in triplicate, and data are expressed as the mean±SEM, *p<0.05, **p<0.01.

To gain insight into the molecular mechanisms by which miR-150 inhibits autophagic flux, TargetScan bioinformatic method was performed to predict the putative targets of miR-150. Among the targets, autophagy-related genes were of particular interest. A preliminary screening identified *EPG5* as a potential miR-150 target (Figure 3A), as EPG5 harbored a miR-150 binding site and was reported to act as a tethering factor mediating the fusion of autophagosomes with lysosomes 13. To validate whether EPG5 is a target gene for miR-150, we generated wild-type (WT) luciferase reporter constructs that included the 3'-UTR of the EPG5 genes and mutant type (MUT) reporter constructs, which contained mutated binding sequences for miR-150 (Figure 3A). Cells were co-transfected with miR-150 and the 3'-UTR constructs to determine the impact of miR-150 to the 3'-UTR of the EPG5 gene. As shown in Figure 3B, compared with the cells transfected with the control vector, co-transfection with miR-150 significantly decreased the luciferase activity from WT 3'-UTR construct, but not from MUT 3'-UTR construct. Furthermore, qRT-PCR and western blotting showed that overexpression of miR-150 profoundly down-regulated EPG5 at mRNA and protein levels in NSCLC cells (Figure 3C-D), while inhibition of miR-150 by miR-150 inhibitor up-regulated the expression of EPG5 in NSCLC cells (Figure 3E). The overexpression and inhibition efficiency of miR-150 were evaluated by qRT-PCR (Figure S3A). Consistent with these *in vitro* results, miR-150 over-expressed tumors derived from mice also showed down-regulated EPG5 protein level (Figure S3B). Notably, while our previous study showed that miR-150 was over-expressed in NSCLC cell lines compared to the MRC-5 normal human lung cells 18, here, in contrast, we detected that EPG5 protein and mRNA levels were significantly down-regulated in NSCLC cell lines (95C, 95D, H1299, H460 and A549) compared with MRC-5 cells (Figure 3F-G). In addition, exogenous overexpression of miR-150 showed more potent inhibitory effect on EPG5 protein level than other potential up-stream miRNAs of EPG5 (miR-9-5p, miR-19-3p, miR-23-3p, miR-30-5p, and miR-143-3p, predicted by TargetScan, Figure S3C). Hence, our results suggested EPG5 was a plausible target of miR-150.

### Silencing EPG5 inhibited autophagic flux and promoted cell growth in NSCLC

**Figure 4:**
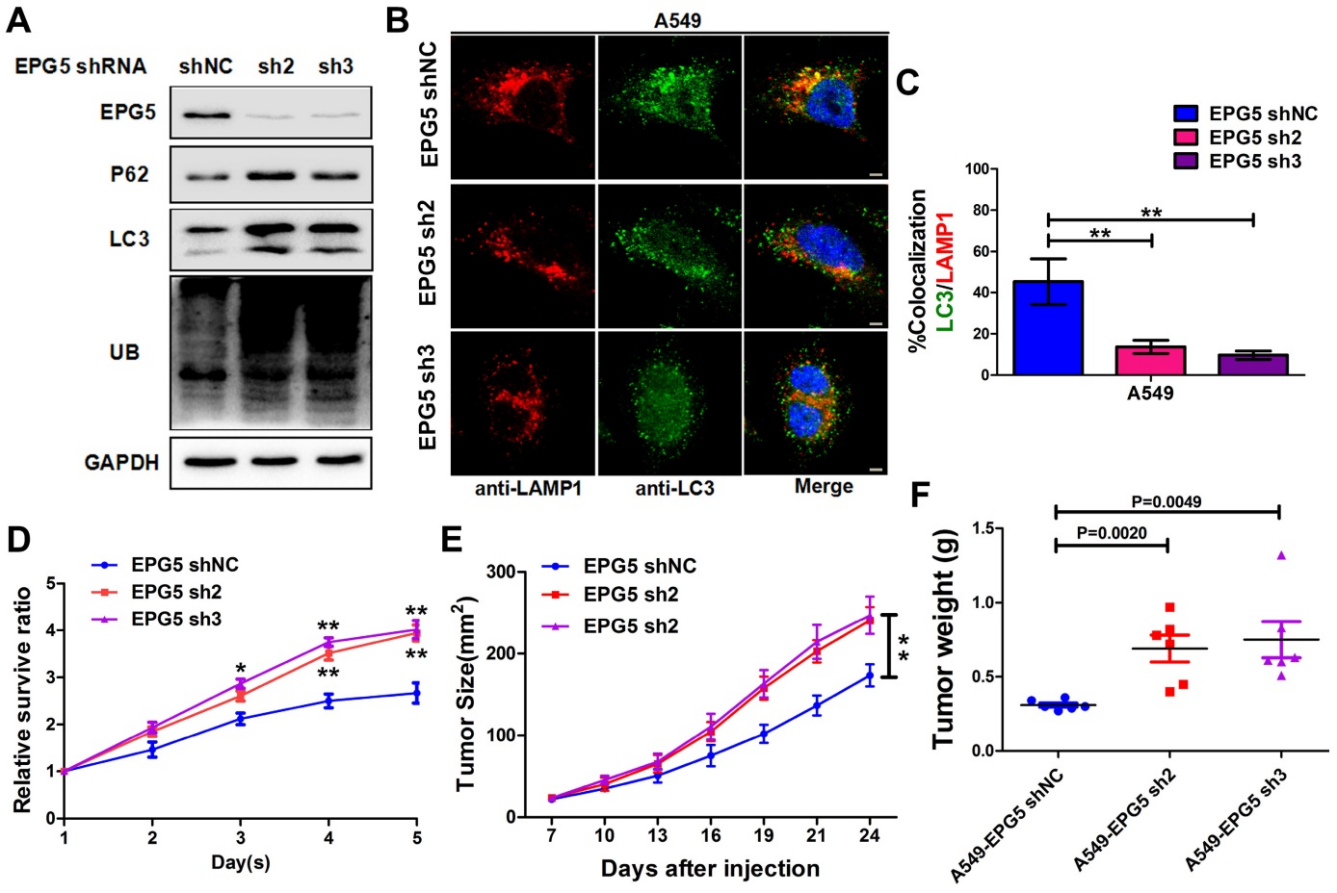
** Silencing EPG5 inhibited autophagic flux and promoted cell growth in NSCLC.** (A) A549 cells stably expressing EPG5-shRNA2 and EPG5-shRNA3 were generated, and the expression levels of EPG5, p62, LC3, UB and GAPDH in control and EPG5-silenced A549 cells were determined by western blotting. Data are representatives of three independent assays. (B) Colocalization of LC3 and LAMP1 was detected by immunofluorescence. Scale bar, 10 μm. Data are representatives of three independent assays. (C) The LC3 and LAMP1 colocalization coefficiency is expressed as mean±SEM, n= ~30 to 50 cells of three independent experiments. (D) Control and EPG5-silenced A549 cells proliferation as measured by the MTT assay. Each bar represents the mean ±SEM of three independent experiments. *p<0.05, **p<0.01. (E) A549 cells expressing control-shRNA, EPG5-shRNA2 or EPG5-shRNA3 were injected subcutaneously into the right flank of the BALB/c nude mice. Data are mean volumes ±SEM at indicated times (n=6 per group). (F) Data are mean weight ±S.E.M of tumor weight. n=6 per group. *p<0.05, **p<0.01.

EPG5 is essential for autophagosome maturation 27, and our results showed its mRNA and protein levels were significantly down-regulated in NSCLC cell lines (Figure 3E-F), therefore we next examined the function of EPG5 on NSCLC cell autophagic flux and tumorigenesis. Firstly, we established two EPG5 stably knockdown cell lines in A549 cells (named as EPG5-sh2 and EPG5-sh3) and a control cell line (named as EPG5-shNC) using two EPG5 short hairpin RNAs (shRNAs) and a control shRNA (Figure 4A, Figure S4). As shown in Figure 4A, western blotting revealed that silencing of EPG5 dramatically increased the accumulation of p62, LC3 and ubiquitinated protein aggregates. Meanwhile, immunofluorescence staining showed silencing of EPG5 led to a significant reduction of co-localization of LC3-labeled autophagosomes with LAMP1-labeled lysosomes (Figure 4B-C). On the other hand, silencing of EPG5 significantly promoted cell proliferation and tumorigenesis *in vitro* and *in vivo* (Figure 4D-E). The volume (Figure S5A) and weight (Figure 4F) of EPG5-silenced tumors were markedly bigger compared with the tumors formed by control cells. Of note, the mice body weight was not significantly different in EPG5 silenced groups and control group (Figure S5B). Together, these results indicated a protective role of EPG5 against NSCLC progression.

### miR-150 inhibited the autophagic flux and promoted NSCLC tumorigenesis through repressing EPG5

**Figure 5:**
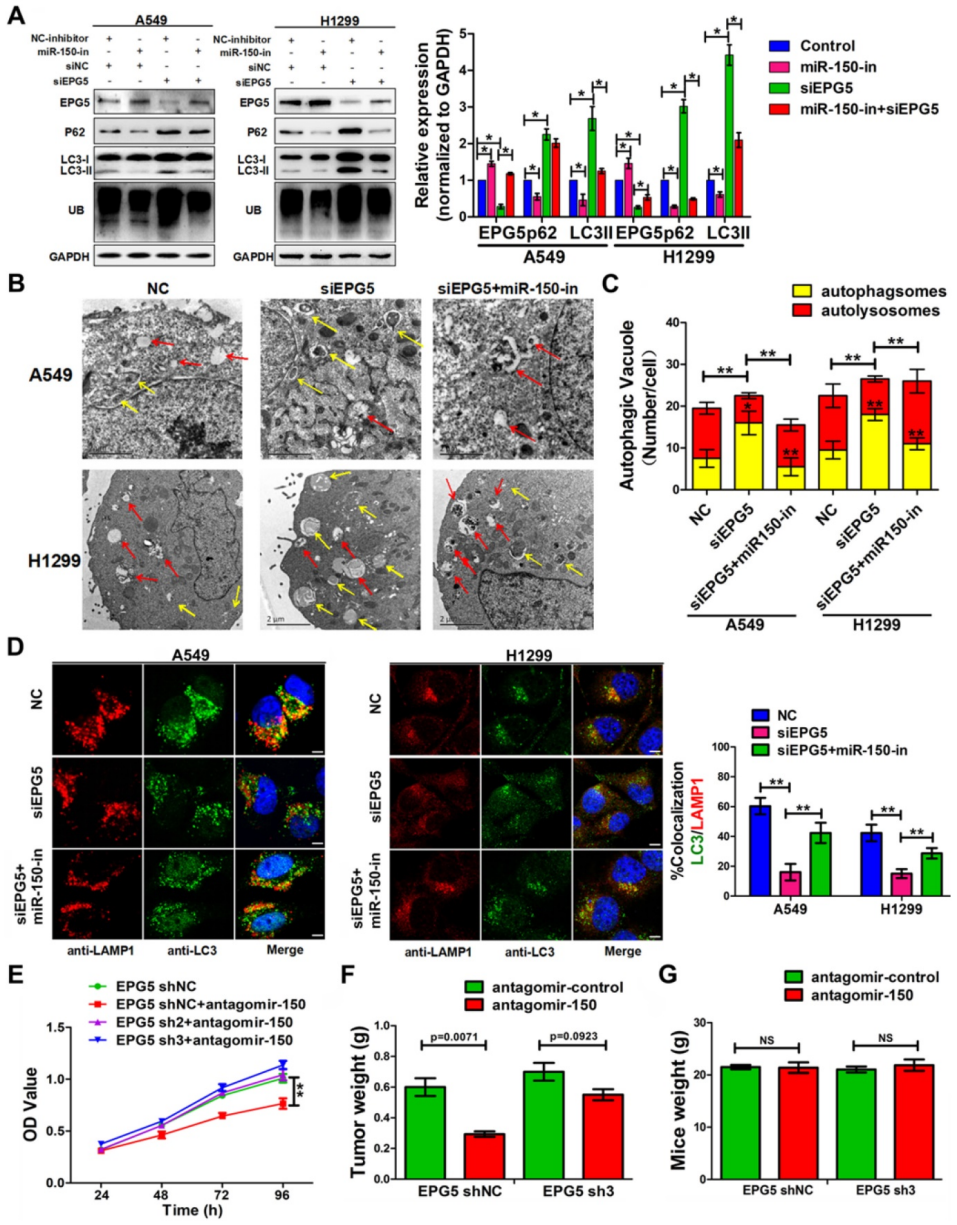
** miR-150 inhibited the autophagic flux and promoted NSCLC tumorigenesis through repressing EPG5.** (A) Western blotting analyses of EPG5, p62, LC3, UB and GAPDH expression in A549 and H1299 cells transfected with NC-inhibitor plus siNC, miR-150 inhibitor plus siNC, NC-inhibitor plus siNC or miR-150 inhibitor plus siEPG5. Images are representatives and bar graphs are quantified results of three independent experiments expressed as the mean±SEM, *p<0.05 compared to the control. (B) A549 and H1299 cells transfected with siNC, siEPG5 or siEPG5 plus miR-150 inhibitor, data are representative images of TEMs of three independent assays. Scale bar, 2 μm. (C) Quantification of autophagosomes (yellow array) and autolysosomes (red array) per cell are expressed as mean±SEM, n= ~20 to 40 cells per group, *p<0.05, **p<0.01. (D) Colocalization of LAMP1 and LC3 was detected by immunofluorescence in A549 and H1299 cells transfected with siNC, siEPG5 or siEPG5 plus miR-150 inhibitor. Scale bar, 10 μm. Data are representatives of three assays. The LC3 and LAMP1 colocalization coefficiency is expressed as mean±SEM, n= ~30 to 50 cells of three independent experiments. (E) The proliferation of control and EPG5-silenced A549 cells treated with antagomir-150 were measured by the MTT assay. Each bar represents the mean ±SEM of three independent experiments. (F, G) A549 cells expressing control-shRNA or EPG5- shRNA3 were injected subcutaneously into the right flank of the BALB/c nude mice, and tail intravenous injection with antagomiR-150 or control. Data are mean weight ±SEM of tumour (F) and mice body (G). n=3 per group. *p<0.05, **p<0.01.

To validate that EPG5 is a functional target of miR-150 and is responsible for inhibiting autophagic flux and inducing cell growth, EPG5 siRNA and miR-150 inhibitor were introduced alone or simultaneously into NSCLC cells. Inhibition of miR-150 by miR-150 inhibitor up-regulated endogenous EPG5 expression, and restored the effect of EPG5 knockdown-induced accumulation of p62, LC3 and ubiquitinated protein aggregates (Figure 5A). Concurrently, inhibition of miR-150 also reversed the autophagic flux-inhibiting effects mediated by EPG5 siRNA, as electron microscope screening revealed the increase number of autolysosomes and less autophagosomes following simultaneous depletion of EPG5 and miR-150 (Figure 5B-C). These results were further strengthened by the observation from immunofluorescence staining that showed increased co-localization of LC3 labeled autophagosome with LAMP1 labeled lysosome after the co-treatment compare to knockdown of EPG5 only (Figure 5D).

On the other hand, miR-150/EPG5 regulation axis was tested as a potential NSCLC therapeutic target. Using antagomir-150, a chemically stabilized miR-150 inhibitor, markedly inhibited control cells; while silencing EPG5 attenuated the anti-tumor effect of antagomir-150 in shEPG5 cells *in vitro* (Figure 5E). Next, subcutaneous xenografts were established in the flanks of nude mice using EPG5-sh3 cells and EPG5-shNC cells. Tail intravenous injection with antagomir-150 significantly decreased the expression of miR-150 in tumors (Figure S5C), and effectively inhibited the growth of EPG5-shNC cells. However, the phenomenon of significant reduction of tumor weights (*p*=0.0071) by antagomir-150 was diminished in EPG5-sh3 cells (*p*=0.0923, Figure 5F, Figure S5D). On the contrary, tail intravenous injection of agomir-150, a chemically stabilized miR-150 mimic, dramatically increased miR-150 expression in tumors (Figure S5C), and notably promoted tumor growth in EPG5-shNC harboring mice without causing any change in EPG5-sh3 xenograft (Figure S5E-G). The mice body weight did not significantly differ among these experimental groups indicating no sign of toxicity for antagomir-150 and agomir-150 treatment (Figure 5G, Figure S5H). These results suggested a reciprocal role of miR-150 and EPG5 during NSLC development.

### C-myc promoted expression of miR-150 and NSCLC tumorigenesis and progression

**Figure 6:**
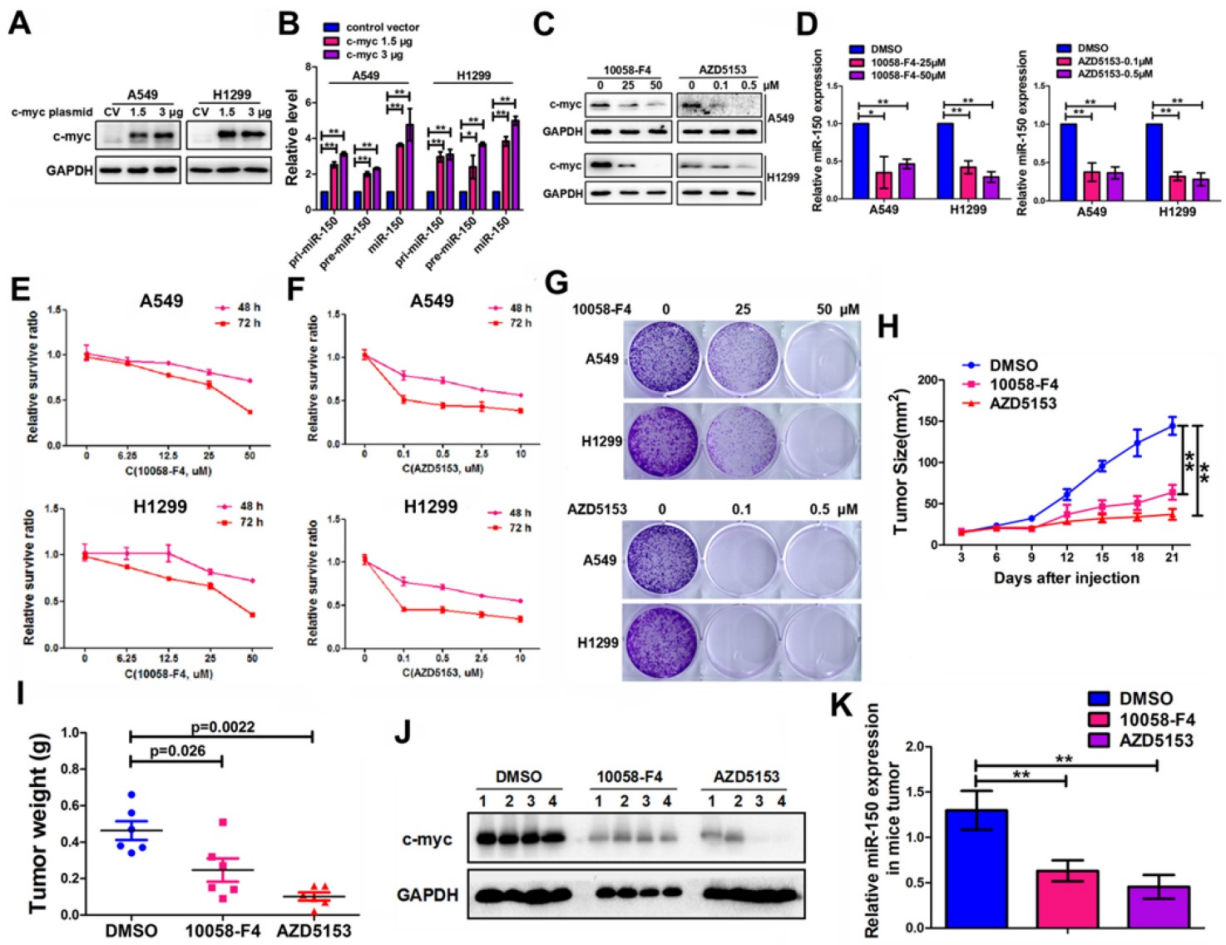
** C-myc promoted expression of miR-150 and NSCLC tumorigenesis and progression.** (A) A549 and H1299 cells transfected with pENTER-c-myc or control vector, c-myc and GAPDH were detected by western blotting. Images are representatives of three independent experiments. (B) The levels of pri-miR-150 (primary miRNA), pre-miR-150 and miR-150 were measured in the control or c-myc overexpressing A549 and H1299 cells by qRT-PCR. Each bar represents the mean ±SEM of three independent experiments. (C) A549 and H1299 cells treated with 10058-F4 and AZD5153 for 48 hours as indicated concentration and c-myc and GAPDH were detected by western blotting. Images are representatives of three independent experiments. (D) A549 and H1299 cells treated with 10058-F4 (left) and AZD5153 (right) for 48 hours as indicated concentration and miR-150 expression were detected by qRT-PCR, U6 serve as an internal control, each bar represents the mean±SEM of three independent experiments. (E, F) The proliferation of A549 and H1299 cells treated with 10058-F4 (E) and AZD5153 (F) for 48 hours (upper) and 72 hours (down) as indicated concentration measured by the MTT assay. Each bar represents the mean±SEM of three independent experiments. (G) The clonality of A549 and H1299 cells measured by the plate clone formation assay after treated with 10058-F4 and AZD5153 for 7 days. (H) A549 cells were injected subcutaneously into the right flank of the BALB/c nude mice, and intraperitoneal injection with 10058-F4 (20mg/kg), AZD5153 (10mg/kg) or DMSO. Data are mean volumes±SEM at indicated times, n=6 per group. (I) Data are mean weight±SEM of tumour. n=6 per group. (J) Proteins extracted from tumor tissues, and c-myc and GAPDH were detected by western blotting. Images are representatives of three independent experiments. (K) RNA extracted from tumor tissues, and miR-150 expression were detected by qRT-PCR, U6 serve as an internal control, each bar represents the mean ±SEM of three independent experiments.*p<0.05, **p<0.01.

In order to understand the underlying mechanism for abnormally high expression of miR-150 in NSCLC, we used a publicly available ChIP-Seq data from the UCSC Genome Browser (*http://genome.ucsc.edu/index.html*) to identify potential transcription factors that are important for miR-150 biosynthesis. Proto-oncogene c-myc was identified as it could potentially bind to the upstream regulatory region of the miR-150 gene (Figure S6). Furthermore, we demonstrated that c-myc bound directly to the upstream region of the miR-150 locus through a chromatin immunoprecipitation (ChIP) assay (Figure S7). To determine any effect of c-myc on miR-150 transcription and maturation in NSCLC, pri-miR-150 (primary miRNA), pre-miR-150 (precursor miRNA) and miR-150 levels were measured in the c-myc-overexpressing cells. As shown in Figure 6A-B, the levels of miR-150 primary, precursor transcripts and mature miR-150 were all increased 2-5 folds when A549 and H1299 cells were transfected with pENTER-c-myc plasmid.

Considering the oncogenic roles of miR-150 in NSCLC and the role of c-myc in miR-150 regulation, we hypothesized that pharmacological inhibition of the expression and/or transcriptional activity of c-myc may be a promising anti-NSCLC therapeutic method. Therefore, we employed c-Myc and BRD4 (Bromodomain-containing protein 4, which transcriptional activates c-myc 28) inhibitors (10058-F4 29 and AZD5153 28). As shown in the results, the level of c-myc in A549 and H1299 cells were dose dependently decreased when treated with c-Myc inhibitor 10058-F4 or BRD4 inhibitor AZD5153 (Figure 6C). These treatments also led to a concomitant decrease in the level of miR-150 (Figure 6D) and increase in the level of EPG5, as well as disordered ER stress and DNA damage pathways (Figure S8). MTT assay and colony formation assay showed that both 10058-F4 and AZD5153 dramatically inhibited cell survival and colony formation *in vitro* (Figure 6E-G). Furthermore, the *in vivo* xenografts experiment showed that compared to DMSO control group, the growth of xenograft tumor cells were significantly inhibited (Figure 6H), and the volume and weight of the tumors were markedly smaller after injecting 10058-F4 and AZD5153 (Figure S9A, Figure 6I). The mice weight did not change in the 10058-F4 group but decreased slightly in AZD5153 group when compared with the control group (Figure S9B). In addition, the western blotting and qRT-PCR results showed that both 10058-F4 and AZD5153 effectively inhibited the expression of c-myc and miR-150 in mice tumor tissues (Figure 6J-K). Taken together, these results suggested that the c-myc promoted NSCLC tumor growth *in vitro* and *in vivo*, and pharmacological inhibition of c-myc/miR-150 pathway would be a promising anti-cancer therapy for NSCLC.

### C-myc/miR-150/EPG5 axis determine prognosis and overall survival of NSCLC patients

**Figure 7:**
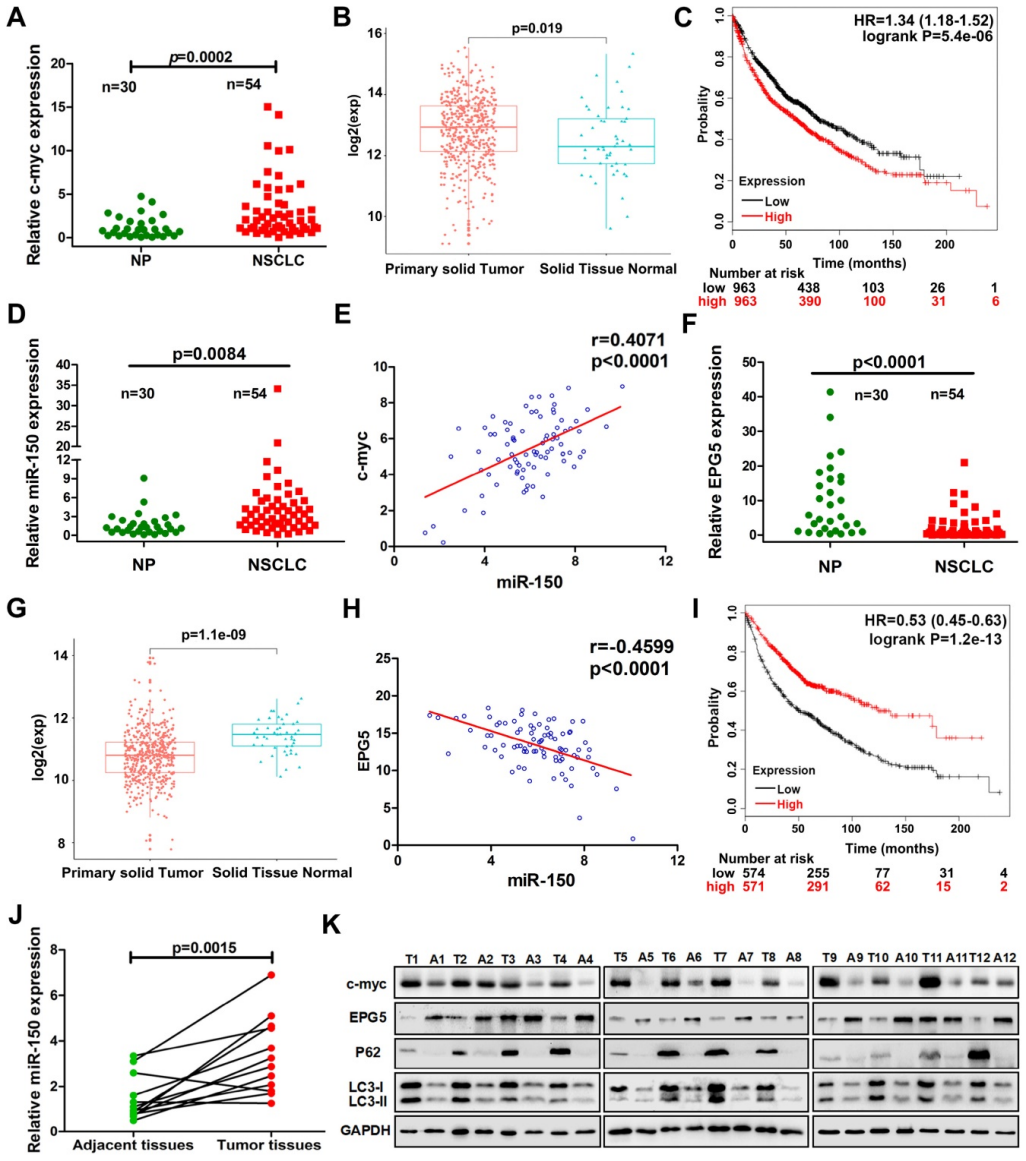
** Correlation of c-myc/miR-150/EPG5 and other key genes with NSCLC development in clinical patients.** (A) Levels of c-myc were measured in human NSCLC tissues (NSCLC, n=54) and non-neoplastic lung tissues (NP, n=30) using qRT-PCR. (B) The expression of c-myc in NSCLC primary solid tumor and solid normal tissue in TCGA data set. (C) Kaplan-Meier survival analysis of overall survival of 3021 NSCLC patients on the basis of c-myc expression. Log-rank test was used to calculate P values. (D) Levels of miR-150 were measured in human NSCLC tissues (NSCLC, n=54) and non-neoplastic lung tissues (NP, n=30) using qRT-PCR. (E) Correlation between miR-150 and c-myc was determined using Spearman coefficient analysis in indicated clinical samples (n=84). (F) Level of EPG5 was measured in human NSCLC tissues (NSCLC, n=54) and non-neoplastic lung tissues (NP, n=30) using qRT-PCR. (G) The expression of c-myc in NSCLC primary solid tumor and solid normal tissue in TCGA data set. (H) Correlation between miR-150 and EPG5 was determined using Spearman coefficient analysis in indicated clinical samples (n=84). (I) Kaplan-Meier survival analysis of overall survival of 1882 NSCLC patients on the basis of EPG5 expression. Log-rank test was used to calculate P values. (J) Level of miR-150 was measured in 12 pairs of NSCLC tissues and adjacent tissues using qRT-PCR. (K) Expression of c-myc, EPG5, p62, LC3, γ-H2AX and GAPDH were measured in 12 pairs of NSCLC tissues and adjacent tissues by western blotting.

To define the clinical relevance of our findings, we examined c-myc, miR-150 and EPG5 expression using qRT-PCR in human normal lung tissues (NP, n=30) and NSCLC biopsy tissues (NSCLC, n=54). Indeed, c-myc expression was significantly higher in NSCLC tissues than in NP tissues (Figure 7A). Meanwhile, the result from TCGA data set showed that the expression of c-myc was markedly higher in NSCLC primary solid tumor than solid normal tissue (Figure 7B). KM-plotter database showed that higher expression of c-myc was significantly associated with shorter survival (Figure 7C). miR-150 also significantly up-regulated in NSCLC tissues than in NP tissues as well as positively correlated with c-myc expression, with Pearson's correlation coefficient of 0.407, *p*<0.0001 (Figure 7D-E). However, EPG5 expression was significantly lower in NSCLC as analyzed in our clinical samples and in TCGA data set (Figure 7F-G). Moreover, EPG5 levels showed significantly negative correlation with miR-150 expression (r=-0.46, p<0.0001, Figure 7H). At the same time, lower level of EPG5 expression was significantly correlated with lower survival of lung cancer patients, which was analyzed in KM-plotter database (Figure 7I).

In addition, we examined the expression of c-myc/miR-150/EPG5 axis related genes in 12 pairs of NSCLC tissues and adjacent tissues. As shown in Figure 7J, compared with the adjacent tissues, miR-150 was significantly up-regulated in NSCLC tissues. Western blotting (Figure 7K) and immunohistochemical staining (Figure S10) showed an obvious increase of c-myc, p62, LC3 and γ-H2AX levels, whereas EPG5 expression was lower in NSCLC tissues compared with the adjacent tissues. Taken together, these results supported that abnormally expressed c-myc/miR-150/EPG5 axis caused dysfunction of authophagy correlated with the NSCLC development.

## Discussion

The role of miR-150 in NSCLC is controversial 18, 30-32, and the precise functions and underlying molecular mechanisms of miR-150 in the context of NSCLC were not clearly defined. In this study, we indicated that miR-150 significantly over-expressed in NSCLC patient tissues, and over-expression of miR-150 promoted the proliferation of NSCLC cells *in vitro* and *in vivo*. Our data suggested that, upon transcriptional activation by c-myc, miR-150 inhibited autophagic flux by repressing EPG5 to induce NSCLC development. At the same time, treatment with 2'-O-methyl-modified miRNA inhibitor (antagomiRNA) 33, 34 targeting miR-150 markedly inhibited the growth of NSCLC *in vivo* and *in vitro*. Thus, our results revealed a previously unknown oncogenic function of miR-150 in NSCLC by modulating autophagy and provided the evidence that c-myc/miR-150/EPG5 axis could be a potential therapeutic target in the clinical setting.

Mounting evidences have supported that dysfunction of autophagy results in a variety of human diseases including cancers 35, 36. Studies from us and others indicated that miR-150 and two key autophagy-relative genes, p62 and LC3 were up-regulated in NSCLC, and both significantly correlated with the tumor progression and poor prognosis 18, 21, 37. Therefore, we subjected to explore whether the miR-150-regulated impaired autophagy further promots NSCLC development. Herein, our results showed that miR-150 apparently blocked the fusion of autophagosome and lysosome, and elevated the accumulation of LC3 and p62. Previous studies have shown that cells with defective autophagic apparatus accumulate damaged organelles, elevated ROS production and activated DNA damage response, and potentially primed cells for tumor development 38, 39. As an adaptive response such as autophagy deficiency, cells up-regulate the expression of GRP78/BiP, a major ER chaperone 40. The ER stress-activated protein kinase PERK phosphorylates the alpha subunit of eukaryotic initiation factor 2 (eIF2α) in stressed cells, which in turn further selectively increases translation of Activating Transcription Factor 4 (ATF4), resulting in the induction of the downstream gene CHOP 41. As shown in our data, overexpression of miR-150 dramatically elevated levels of Bip, p-eIF2α, ATF4 and CHOP, indicating that miR-150-mediated autophagy defects induce ER stress. Furthermore, overexpression of miR-150 increased cellular ROS levels and DNA damage response. Taken together, the accumulation of p62, ER stress, ROS and DNA damage resulting from miR-150-mediated autophagy defect may result in enhancement of mutations, genome instability and NSCLC tumorigenesis and progression.

Subsequently, we investigated the underlying molecular mechanism of miR-150 and revealed that miR-150 blocks the fusion of autophagosome with lysosome by directly binding to the 3'-UTR of EPG5, an autophagosome maturation-related protein 13, and repressing its mRNA and protein expression. Interestingly, we found that EPG5 expression was significantly lower in NSCLC, and significantly negatively correlated with miR-150 expression. Furthermore, the expression of EPG5 was correlated with longer survival of lung cancer patients and silencing EPG5 significantly promoted NSCLC cells growth, and attenuated the pro-tumor growth effect of miR-150* in vitro* and *in vivo*. Therefore, we defined EPG5 as a new functional target of miR-150 and as a novel NSCLC tumor-suppressor.

Finally, we explored the molecular mechanism that why miR-150 up-regulated in NSCLC. Previous studies reported that transcription factor c-myc is an important regulator of miRNAs biosynthesis, including transcription and maturation 42. C-myc could bind to the promoter region and activates or represses the transcript of miRNAs, such as miR-17, miR-9 or miR-34a and miR-26a 43. Meanwhile, c-myc has been reported to play a critical role in malignant transformation and is the most frequently amplified oncogene in human cancers, including breast, colon and lung cancer 44. In this study, we identified that c-myc directly bound to the upstream region of the miR-150 and as an upstream regulator for miR-150 gene transcription. Overexpression of c-myc promoted the transcription and maturation of miR-150 in NSCLC cells. Meanwhile, our results demonstrated that c-myc was significantly highly expressed in NSCLC and positively correlated with miR-150 expression, and both c-myc and miR-150 significantly associated with shorter survival. Furthermore, pharmacological inhibition of c-myc significantly repressed the expression of miR-150 and the proliferation of NSCLC cells *in vitro* and *in vivo*. Considering the important roles of miR-150 in NSCLC tumorigenesis and development, potentially therapeutic use of chemically stabilized miR-150 inhibitor will be a replacement therapy. However, limited by the high cost of chemical modification and “off target” effects of miRNA inhibitors, continuing development of small molecules, which act pharmacologically to inhibit the c-myc can be a efficient therapeutic strategy for NSCLC 45.

Taken together, in this study, we revealed that high expression of miR-150 notably inhibited the fusion of autophagosomes with lysosomes through repressing the expression of EPG5, which played a tumor-suppressor role in NSCLC cells. C-myc acted as an upstream transcriptional regulator of miR-150 and pharmacological or genetic inhibition of c-myc/miR-150 significantly inhibited NSCLC cell growth *in vitro* and tumor growth *in vivo*. Our study disclosed that c-myc/miR-150/EPG5 axis mediated dysfunction of autophagy induced the accumulation of ER stress, ROS and DNA damage, and promoted the development of NSCLC, which may provide an effective therapeutic target to treat NSCLC.

## Supplementary Material

Supplementary figures and tables.

## Abbreviations

ATG9A: autophagy-related protein 9A
Baf A1: bafilomycin A1
BRD4: Bromodomain-containing protein 4
eIF2α: eukaryotic initiation factor 2
EPG5: Ectopic P granules protein 5 homolog
ER: Endoplasmic Reticulum
LAMP1: lysosome-associated membrane protein 1
NSCLC: non-small cell lung cancer
OPTN: Optic neuropathy-inducing protein
ShRNAs: short hairpin RNAs
TEM: transmission electron microscopy
