# Supplementary material for: C-myc/miR-150/EPG5 axis mediated dysfunction of autophagy promotes development of non-small cell lung cancer
Source: Theranostics. 2019 Jul 9;9(18):5134–48. doi: 10.7150/thno.34887 (PMC6691579; doi:10.7150/thno.34887)
Supplement: Supplementary file 1 — Supplementary figures and tables. [file thnov09p5134s1.pdf]

Supplemental Documents

Table S1. The list of primers and oligomers

Gene	Sequence
EPG5 siRNA sequence	5-GAACAGGUGUACCCAAGCU-3 (sense);
RNA duplex control sequence	5-UUCUCCGAACGUGUCACGUTT-3 (sense);
miR-150 inhibitor sequence	5-CACUGGUACAAGGGUUGGGAGA-3 (sense)
miR-150 inhibitor NC sequence	5-CAGUACUUUUGUGUAGUACAA-3 (sense)
miR-150 mimics NC sequence	5-UUUGUACUACACAAAAGUACUG-3 (sense)
miR-150 mimics sequence	5-GUGACCAUGUUCCCAACCCUCU-3 (sense)
EPG5 primer sequence	5-CCUGGAGUGUGACAUGGAUAATT-3 (sense); 5-UUAUCCAUGUCACACUCCAGGTT-3 (antisense);
P62 primer sequence	5-CGAACGGGATGAATGGAAAG-3 (sense) 5-CGGACAGCGAAAGGTATAAG-3 (antisense);
GAPDH primer sequence	5-CATGAGAAGTATGACAACAGCCT-3 (sense) 5-AGTCCTTCCACGATACCAAAGT-3 (antisense);

Table S2. The information of antibodies

Antibody	Source
Anti-EPG5	38061, Invitrogen
Anti-EPG5	TDY349F, Beiijng TDY Biotech CO., Ltd
Anti-SQSTM1 (Rabbit)	ab109012, abcam
Anti-SQSTM1 (Moues)	sc-28359, Santa Cruz Biotechnology
Anti-LC3 (Rabbit)	ab48394, abcam
Anti-LC3 (Rabbit)	2775S, Cell Signaling Technology
Anti-LC3 (Mouse)	sc-292354, Santa Cruz Biotechnology
Anti-LAMP1 (Mouse)	sc-18821, Santa Cruz Biotechnology
Anti-p53	sc-47698, Santa Cruz Biotechnology
Anti-ubiquitin	ab7780, abcam
Anti-GAPDH	sc-365062, Santa Cruz Biotechnology
Anti-Optineurin	D80D3, Santa Cruz Biotechnology
Anti-Bip	3177T, Cell Signaling Technology
Anti-c-myc	ab117635, abcam
Anti-γ-H2AX	9718S, Cell Signaling Technology
Anti-RRC1	ab117635, abcam
Anti-α-tubulin	ab117635, abcam
Anti-p-EIF2α	3398T, Cell Signaling Technology
Anti-EIF2α	5324T, Cell Signaling Technology
Anti-ATF4	11818S, Cell Signaling Technology
Anti-chop	A0932, Cell Signaling Technology

Supplemental Figure 1

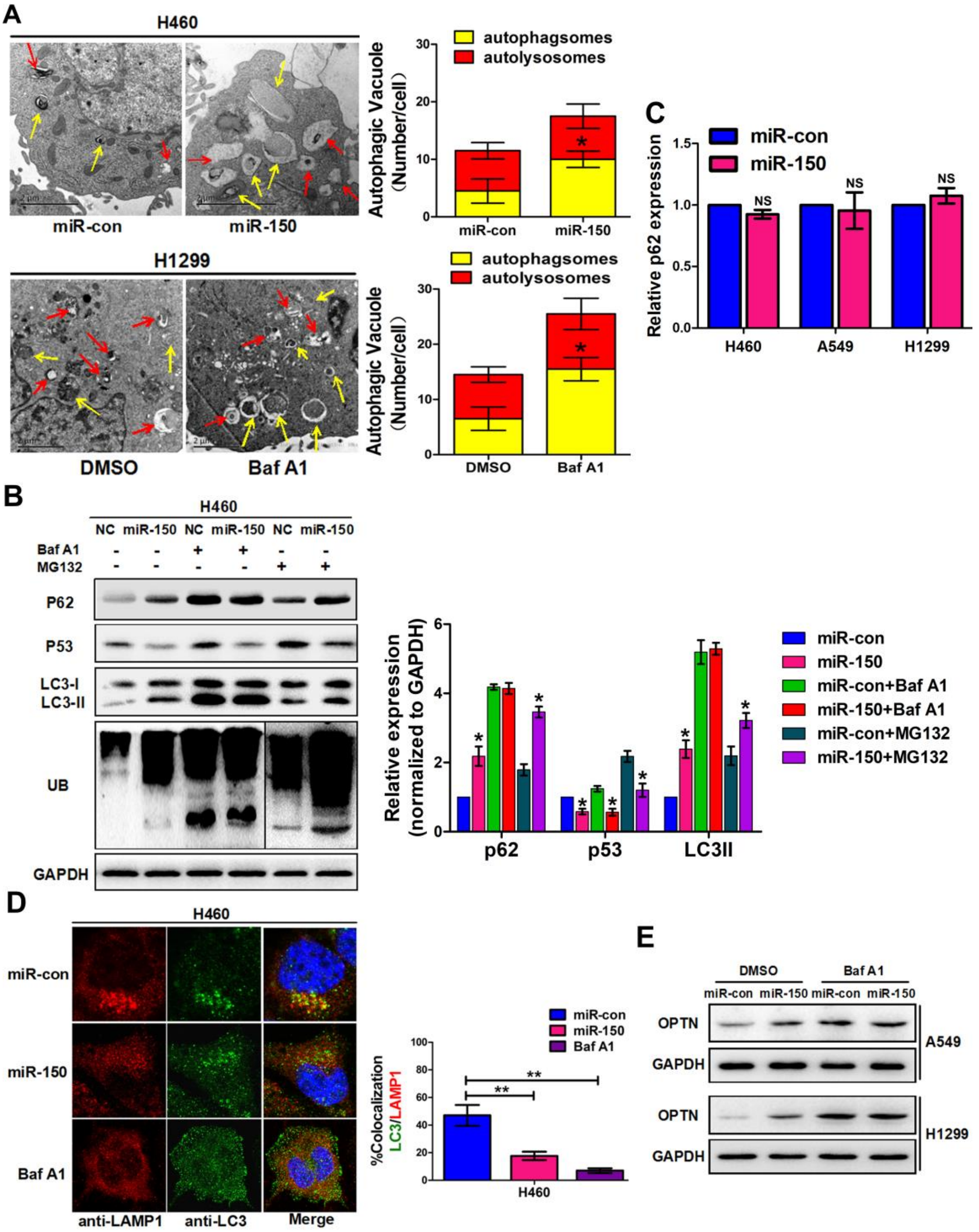

Supplemental Figure 1. miR-150 inhibits the fussion of autophagosomes with lysosomes. (A) Overexpressing miR-150 increased the number of autophagosomes (yellow array) and decreased the number of autolysosomes (red array) in H460 cells. Data are representative images of TEMs of three independent assays. Scale bar, 2 μm. Quantification of autophagosomes and autolysosomes per cell are expressed as

mean±SEM, n= ~20 to 40 cells per group, *$p<0.05$, **$p<0.01$. (B) H460 cells transfected with miR-150 or control vector were treated with Baf A1 (100 nM) and MG132 (10 μM) for 4 h and indicated proteins were detected by western blotting. Images are representatives and bar graphs are quantified results of three independent experiments expressed as the mean±SEM, *$p<0.05$ compared to the control. (C) H460, A549 and H1299 cells transfected with miR-150 or control vector and p62 mRNA expression level were detected by qRT-PCR, data are quantified results of three independent experiments expressed as the mean±SEM, $^{NS}p>0.05$. (D) Colocalization of LC3 and LAMP1 was detected by immunofluorescence. Scale bar, 10 μm. Data are representatives of three independent assays. The LC3 and LAMP1 colocalization coefficiency is expressed as mean±SEM, n= ~30 to 50 cells of three independent experiments. (E) A549 and H1299 cells transfected with miR-con or miR-150 were treated with Baf A1 (100 nM) for 4 h and OPTN protein level was detected by western blotting. Images are representatives of three independent experiments.

Supplemental Figure 2

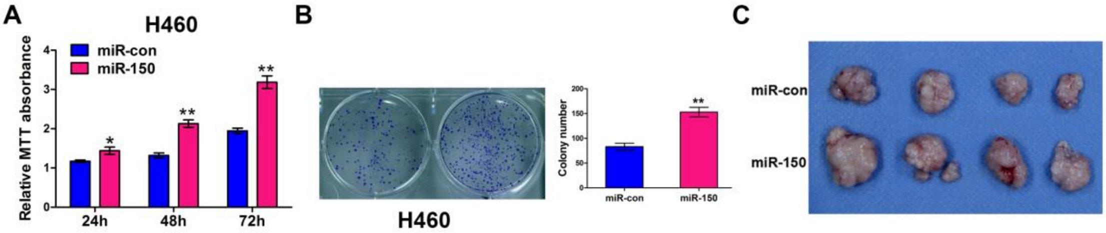

Supplemental Figure 2. miR-150 promoted NSCLC cell proliferation. (A) H460 cells proliferation was measured by the MTT assay after transfected with miR-con or miR-150, data are quantified the results of three independent experiments expressed as the mean±SEM, *p<0.05, **p<0.01. (B) H460 cells clonality was measured by the plate clone formation assay after stably transfected with miR-150 or control vector. Images are representatives and bar graphs are quantified results of three independent experiments expressed as the mean±SEM, **p<0.01 compared to the control. (C) H460 cells stably transfected with miR-150 or control vector were injected subcutaneously into the upper backs of the BALB/c nude mice. Image is the representative xenograft tumors from the mice.

Supplemental Figure 3

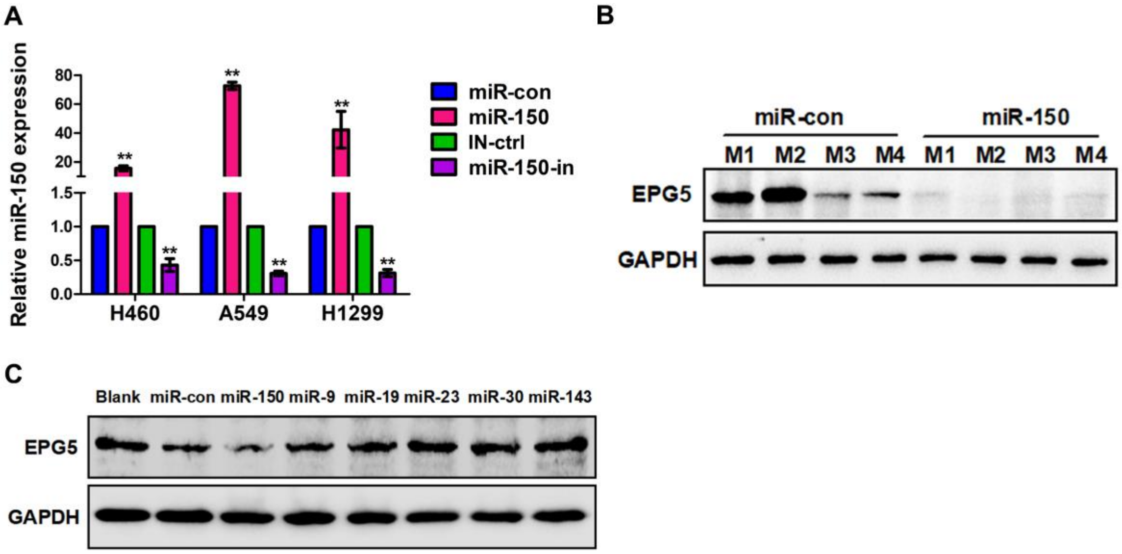

Supplemental Figure 3. EPG5 was a novel target for miR-150. (A) Relative fold changes in overexpression or inhibition of miR-150 by transfection with pri-miR-150 plasmid or miR-150 inhibitor (miR-150-in) respectively were evaluated by qRT-PCR. The expression of miR-150 in the cells transfected with null vectors (miR-con) or inhibitor control (IN-ctrl) was used as control. The results were shown as the mean±SD of three independent experiments, $^{**}p<0.01$. (B) The protein level of EPG5 in miR-150 stable cell line xenograft tumors was detected by western blotting. GAPDH served as control. (C) Western blotting analysis of EPG5 expression in A549 cells transfected with miR-150-5p and other potential up-stream miRNAs of EPG5 (miR-9-5p, miR-19-3p, miR-23-3p, miR-30-5p, miR-143-3p), which were predicted through TargetScan datebase. GAPDH served as the loading control. Images are representatives of three independent experiments.

Supplemental Figure 4

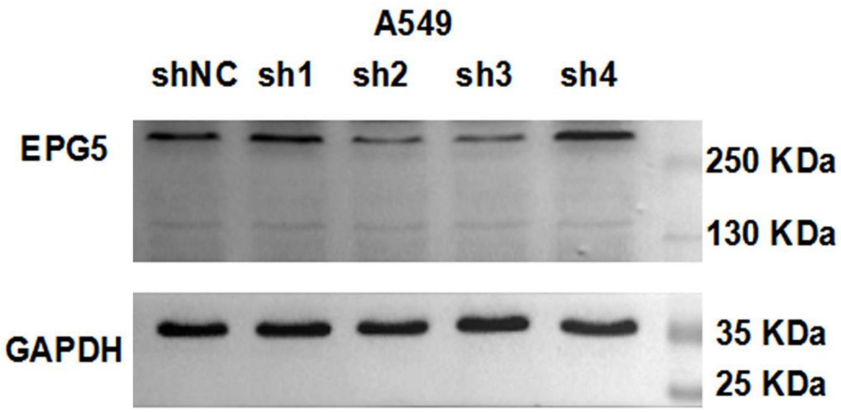

Supplemental Figure 4. Effects of EPG5 shRNAs on the protein expression of EPG5. Western blotting analysis of EPG5 expression in A549 cells transfected with a negative control and four different of EPG5 shRNAs, respectively. GAPDH served as the loading control. Images are representatives of three independent experiments.

Supplemental Figure 5

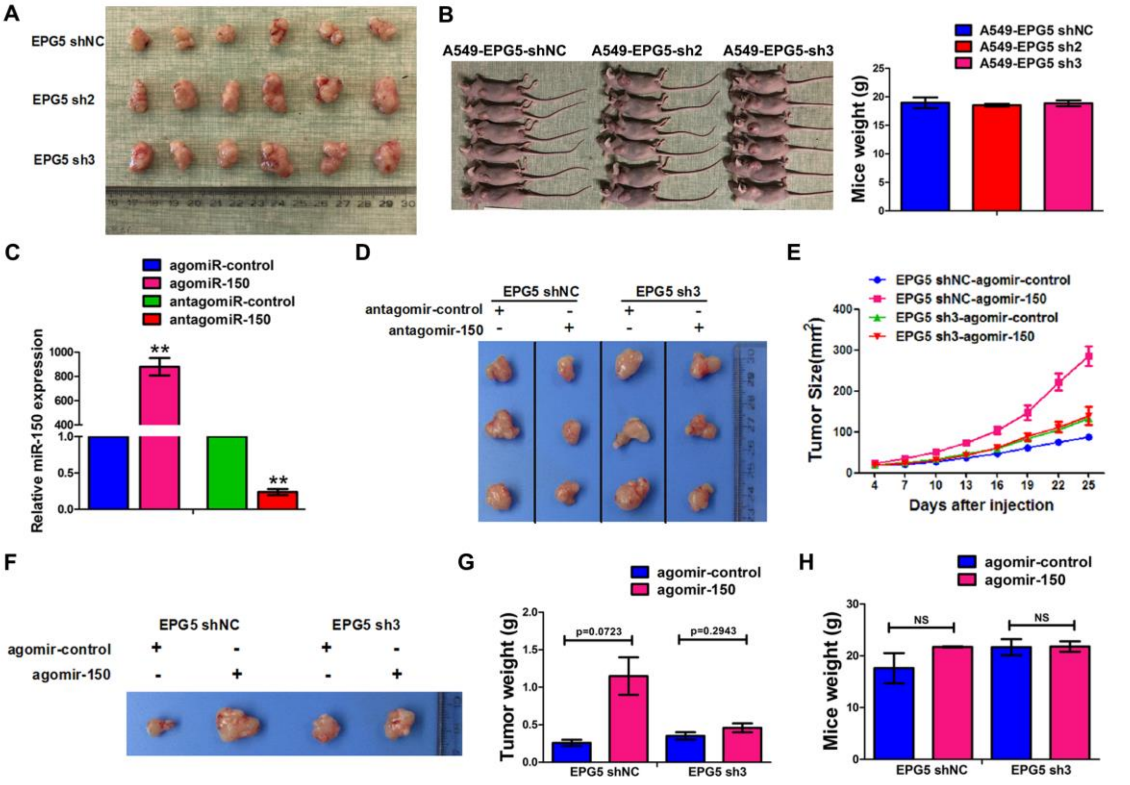

Supplemental Figure 5. Silencing EPG5 impaired miR-150 induced tumorigenesis. (A, B) A549 cells expressing control-shRNA or EPG5-shRNA3 were injected subcutaneously into the right flank of the BALB/c nude mice. Data are photographs of representative tumors (A), mice body and mean weight±SEM of mice body, n=6 per group (B). (C) Extract the RNA from tumors and the expression of miR-150 were measured by qRT-PCR. Data are quantified results of three independent experiments expressed as the mean±SEM, $^{**}p<0.01$. (D) A549 cells expressing control-shRNA or EPG5-shRNA3 were injected subcutaneously into the right flank of the BALB/c nude mice, and tail intravenous injection with antagomiR-150 or control. Data are photographs of representative tumors. (E) A549 cells expressing control-shRNA or EPG5-shRNA3 were injected subcutaneously into the right flank of the BALB/c nude mice, and tail intravenous injection with agomiR-150 or control. Data are mean volumes±SEM at indicated times (n=3 per group). (F) Data are photographs of representative tumors. (G, H) Data are mean weight±SEM of tumour (G) and mice body (H). n=3 per group. $^{*}p<0.05$, $^{**}p<0.01$.

Supplemental Figure 6

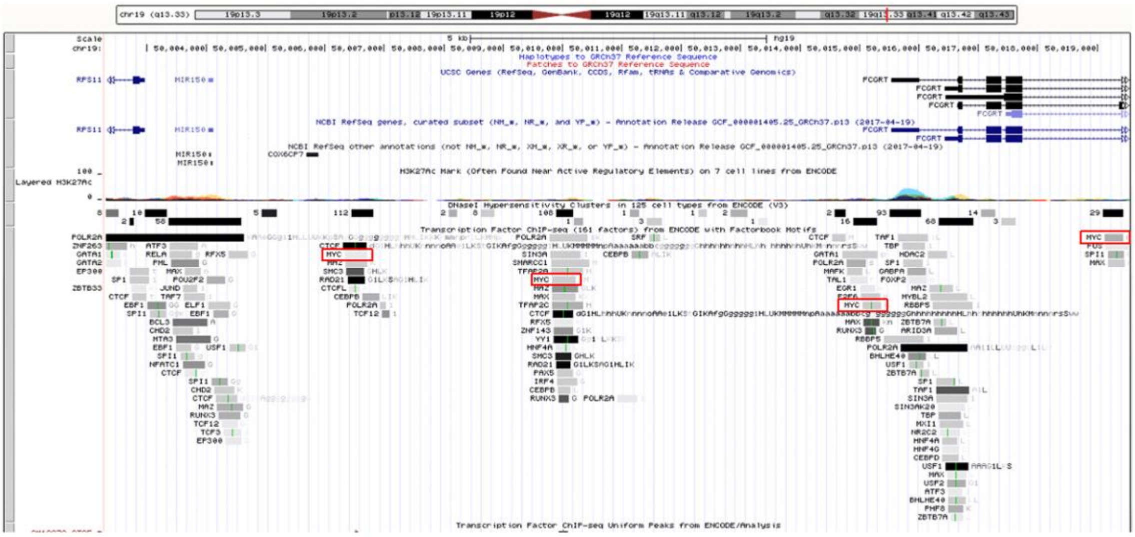

Supplemental Figure 6. Protein binding at miR-150 upstream DNA (ChIP-Seq from UCSC), red empty squares indicate putative myc-binding sites.

Supplemental Figure 7

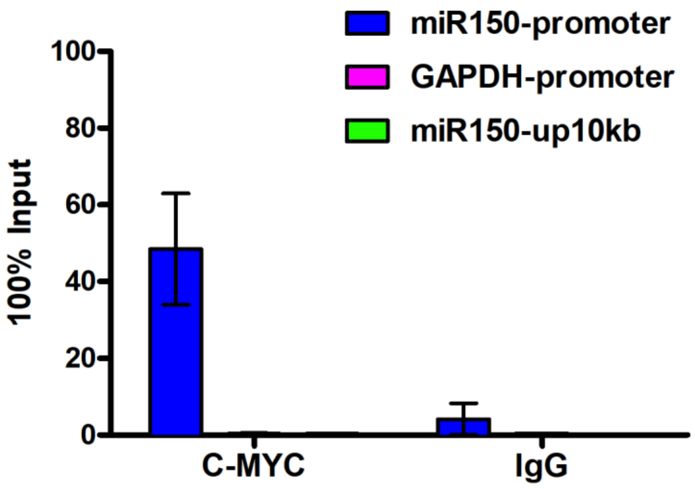

Supplemental Figure 7. ChIP assays of potential binding of c-myc to the miR-150 gene locus in A549 cells. The protein-DNA complexes were precipitated in anti-c-Myc antibody while anti-IgG antibody was used as negative control. The region containing putative c-Myc binding sites of miR-150 promoter were analyzed by qRT-PCR with specific primers, and >10 kb upstream of the miR-150 locus and GAPDH promoter as the control locus.

Supplemental Figure 8

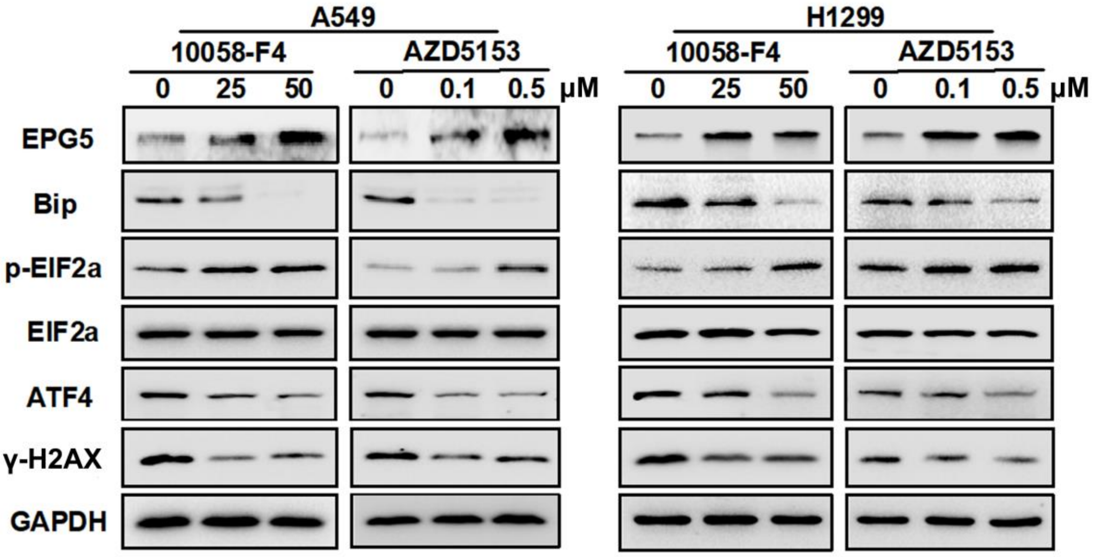

Supplemental Figure 8. A549 and H1299 cells treated with 10058-F4 and AZD5153 for 48 hours as indicated concentration. then, the ER stress-related proteins and γ-H2AX were detected by western blotting. Images are representatives of three independent experiments

Supplemental Figure 9

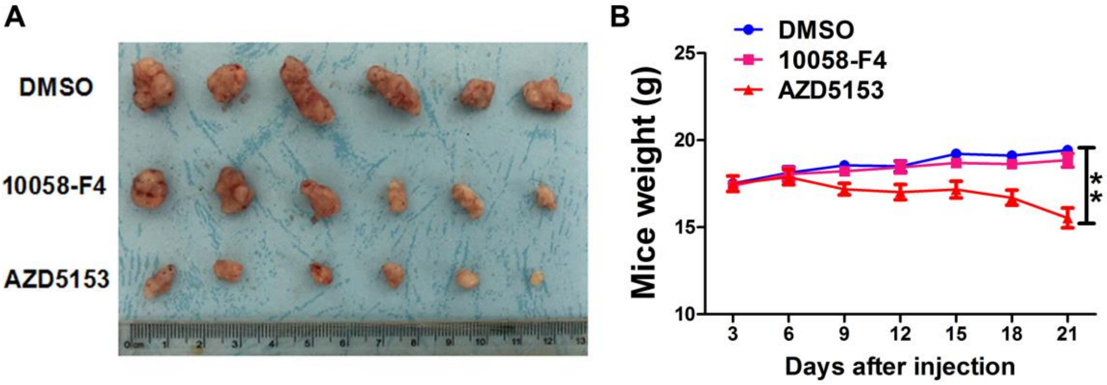

Supplemental Figure 9. Inhibition of c-myc inhibits NSCLC tumor growth *in vivo*. A549 cells were injected subcutaneously into the right flank of the BALB/c nude mice, and intraperitoneal injection with 10058-F4 (20mg/kg), AZD5153 (10mg/kg) or DMSO. (A) Data are photographs of representative tumors. (B) Data are mean weight±SEM of mice body. n=6 per group.

Supplemental Figure 10

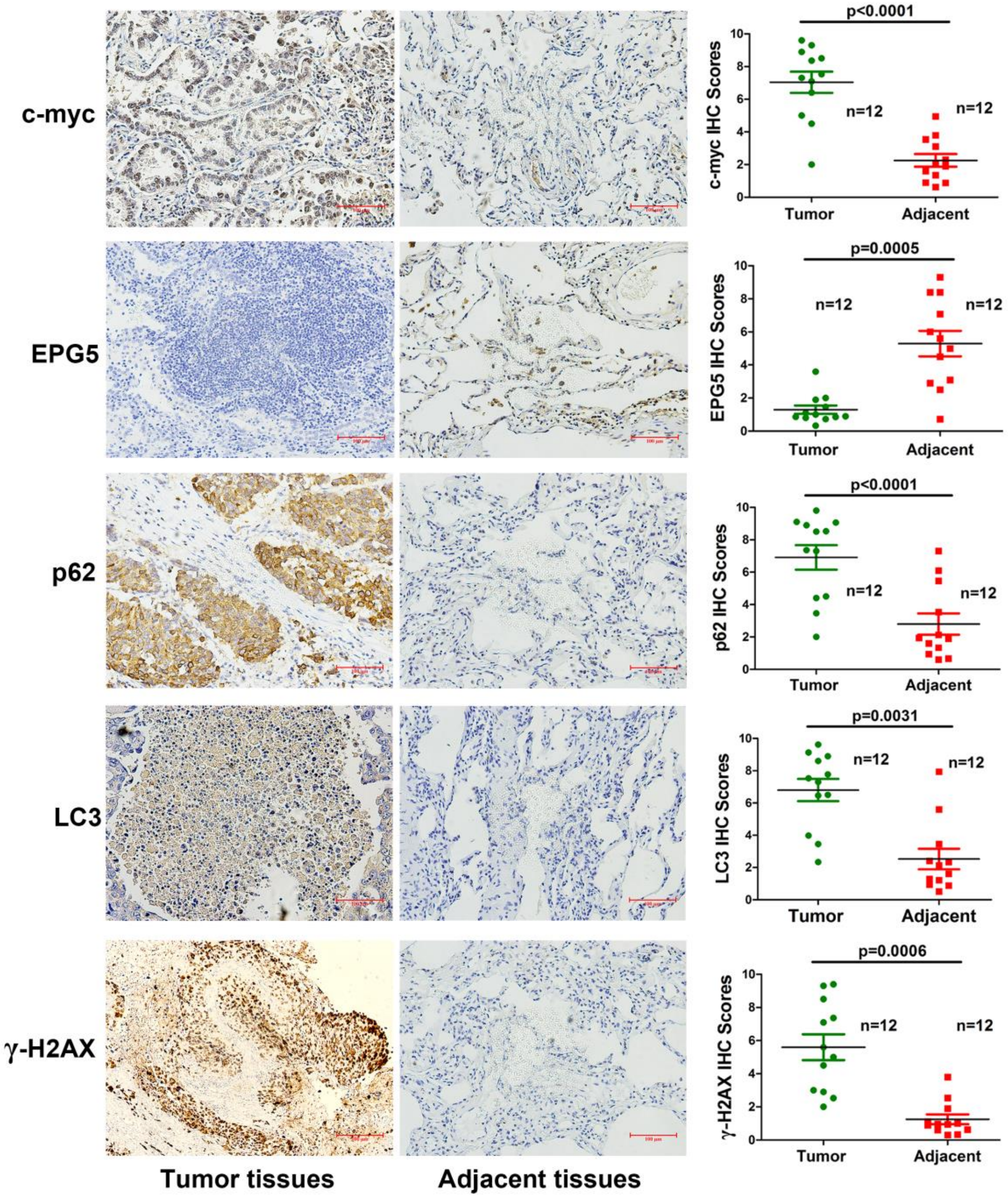

Supplemental Figure 10. The expression of c-myc, EPG5, p62, LC3 and γ-H2AX was detected with immunohistochemical staining in 12 pairs of NSCLC tissues and adjacent tissues. Data are representative of stained tumor and adjacent tissues (left; scale bar, 100 μm) with quantized analyses of paired clinical samples (right). **p*<0.05, ***p*<0.01.
